# Retinal Ganglion Cell Degeneration Linked to Pathogenic Tau Accumulation in Early and Late Stages of Alzheimer's Disease

**DOI:** 10.1002/alz70856_100353

**Published:** 2025-12-25

**Authors:** Dieu‐Trang Fuchs, Yosef Koronyo, Miyah R Davis, Edward K Robinson, Elena Salobrar‐Garcia, Altan Rentsendorj, Bhakta Prasad Gaire, Rakez Kayed, Lon S. S Schneider, Keith L Black, Alfredo A Sadun, Maya Koronyo‐Hamaoui

**Affiliations:** ^1^ Department of Neurosurgery, Maxine Dunitz Neurosurgical Research Institute, Cedars‐Sinai Medical Center, Los Angeles, CA, USA; ^2^ Institute of Ophthalmologic Research Ramón Castroviejo, Complutense University of Madrid, Madrid, Madrid, Spain; ^3^ University of Texas Medical Branch, Galveston, TX, USA; ^4^ Alzheimer's Disease Research Center, Keck School of Medicine, University of Southern California, Los Angeles, CA, USA; ^5^ Doheny Eye Institute, University of California Los Angeles, Los Angeles, CA, USA; ^6^ Department of Ophthalmology, David Geffen School of Medicine at University of California Los Angeles, Los Angeles, CA, USA; ^7^ Department of Neurology, Cedars‐Sinai Medical Center, Los Angeles, CA, USA; ^8^ Department of Biomedical Sciences, Division of Applied Cell Biology and Physiology, Cedars‐Sinai Medical Center, Los Angeles, CA, USA

## Abstract

**Background:**

Histological and in vivo imaging studies suggest a loss of retinal ganglion cells (RGCs) in Alzheimer's disease (AD) patients, potentially contributing to dysfunctions such as contrast sensitivity, color vision, and circadian rhythms. We previously reported elevated pathogenic‐tau isoforms, particularly hyperphosphorylated tau at serine 396 (pS396‐tau) and tau oligomers (oligo‐tau), in the retinas of patients with mild cognitive impairment (MCI, due‐to‐AD) and AD dementia compared to cognitively normal (CN) individuals. However, the presence of pathogenic‐tau isoforms in RGCs and their impact on RGC integrity in AD have not been studied.

**Method:**

Retinal cross‐sections from 25 patients with MCI or AD and 16 age‐ and sex‐matched CN controls were analyzed. Immunofluorescence and Nissl staining were conducted, using the RGC marker ribonucleic acid binding protein with multiple splicing (RBPMS) in conjunction with pathogenic tau‐isoforms, and markers of apoptosis, granulovacuolar degeneration (GVD) bodies, and GVD‐associated necroptosis. Stereological quantifications and analyses of RGC size and distribution were performed. Findings were correlated with corresponding brain pathology and cognitive status.

**Result:**

We present the first evidence of tau inclusions, pS396‐tau and T22^+^ oligo‐tau, within RBPMS^+^ RGCs, alongside a 46–56% reduction in RBPMS^+^‐RGCs and Nissl^+^‐neurons in the ganglion cell layer of MCI and AD retinas. RGC loss was accompanied by morphological and molecular alterations: soma hypertrophy (10‐50% enlargement), nuclear displacement, apoptosis (30‐50% increase), and prominent expression of CHPM2B^+^ GVD bodies and pMLKL^+^ GVD‐necroptotic markers. PS396‐tau^+^‐RGC and oligo‐tau^+^‐RGC counts were 2.1–3.5‐fold higher in MCI and AD retinas compared to CN. Tauopathy‐laden RGCs intercorrelated (*r* =  0.85, *p* <0.0001), and were associated with RGC reduction (*r* =  ‐0.40–(‐0.64), *p* <0.05–0.01). Their abundance linked with advanced brain pathology (Braak stage V–VI) and cognitive decline (clinical dementia rating 3, mini mental state evaluation <26).

**Conclusion:**

Our findings suggest that abnormal tau‐isoforms accumulate within RBPMS^+^ RGCs in MCI and AD patients, contributing to early and pronounced RGC loss and correlating with disease severity. This link involves apoptotic and GVD‐necroptotic cell death pathways. Future research should validate these results in larger cohorts and develop RGC‐tauopathy as a noninvasive biomarker for early AD detection and monitoring.

